# Unique features and clinical importance of acute alloreactive immune responses

**DOI:** 10.1172/jci.insight.97219

**Published:** 2018-05-17

**Authors:** Charlotte F. Inman, Suzy A. Eldershaw, Joanne E. Croudace, Nathaniel J. Davies, Archana Sharma-Oates, Tanuja Rai, Hayden Pearce, Mirjana Sirovica, Y.L. Tracey Chan, Kriti Verma, Jianmin Zuo, Sandeep Nagra, Francesca Kinsella, Jane Nunnick, Rasoul Amel-Kashipaz, Charles Craddock, Ram Malladi, Paul Moss

**Affiliations:** 1 Institute of Immunology and Immunotherapy, College of Medical and Dental Sciences, and; 2 Centre for Computational Biology, Institute of Cancer and Genomic Sciences, College of Medical and Dental Sciences, University of Birmingham, Birmingham, United Kingdom.; 3 Birmingham Health Partners, Department of Haematology, Queen Elizabeth Hospital, Birmingham, United Kingdom.

**Keywords:** Hematology, Immunology, Bone marrow transplantation, T cells

## Abstract

Allogeneic stem cell transplantation (allo-SCT) can cure some patients with hematopoietic malignancy, but this relies on the development of a donor T cell alloreactive immune response. T cell activity in the first 2 weeks after allo-SCT is crucial in determining outcome, despite the clinical effects of the early alloreactive immune response often not appearing until later. However, the effect of the allogeneic environment on T cells is difficult to study at this time point due to the effects of profound lymphopenia. We approached this problem by comparing T cells at week 2 after allograft to T cells from autograft patients. Allograft T cells were present in small numbers but displayed intense proliferation with spontaneous cytokine production. Oligoclonal expansions at week 2 came to represent a substantial fraction of the established T cell pool and were recruited into tissues affected by graft-versus-host disease. Transcriptional analysis uncovered a range of potential targets for immune manipulation, including OX40L, TWEAK, and CD70. These findings reveal that recognition of alloantigen drives naive T cells toward a unique phenotype. Moreover, they demonstrate that early clonal T cell responses are recruited to sites of subsequent tissue damage and provide a range of targets for potential therapeutic immunomodulation.

## Abstract 2

Alloreactive response T cells at 2 weeks after allo-SCT displayed intense proliferation with spontaneous cytokine production, and were recruited into tissues affected by GvHD.

## Introduction

Allogeneic stem cell transplantation (allo-SCT) remains a highly effective therapy for hematopoietic malignancies that are not curable by chemotherapy and/or radiotherapy. The potentially curative nature of SCT is due to generation of a graft-versus-leukemia response, which is mediated by the donor immune system. However, this alloreactive immune response (AIR) can also result in graft versus host disease (GvHD), which remains one of the major causes of morbidity and mortality associated with allo-SCT. T cells are central to the initiation and maintenance of the AIR, and quantitative and qualitative aspects of T cell reconstitution after SCT are strongly linked to clinical outcome (1–3). The initial AIR is mediated by mature T cells transferred within the donor stem cell graft (4), and several studies support the concept that the AIR is generated in the first 2 weeks after transplantation, in line with other primary T cell responses (5–11). In particular, the administration of cyclophosphamide (Cy) at days 3–4 after SCT is effective at reducing the incidence of GvHD and is believed to act through selective depletion of proliferating alloreactive T cells at this very early stage (12–18). An improved understanding of T cell biology during the early AIR in human subjects might allow potential therapeutic intervention to selectively guide T cell alloreactivity toward elimination of host hemopoiesis while minimizing risk of GvHD. However, one of the challenges with the study of AIR in patients following allo-SCT is that transfer of T cells into lymphopenic patients leads to intense homeostatic proliferation, which complicates assessment of the response to antigen-specific allorecognition (19–21).

To isolate the effects of the AIR, we characterized and contrasted T cell phenotype and function at 2 weeks after transplant in allograft and autograft patients. We demonstrate that T cells from patients following allograft are highly activated, incorporated into the long-term T cell repertoire, and are recruited into GvHD-affected tissue. Transcriptional analysis identifies several potential targets for early immunomodulation of the AIR. These data provide a comprehensive characterization of the early human T cell AIR and open avenues of investigation for its potential modulation.

## Results

### Small numbers of T cells are detectable within peripheral blood at 2 weeks after allograft and demonstrate relative expansion of donor CD4 and CD8 effector cells.

**Figure 1:**
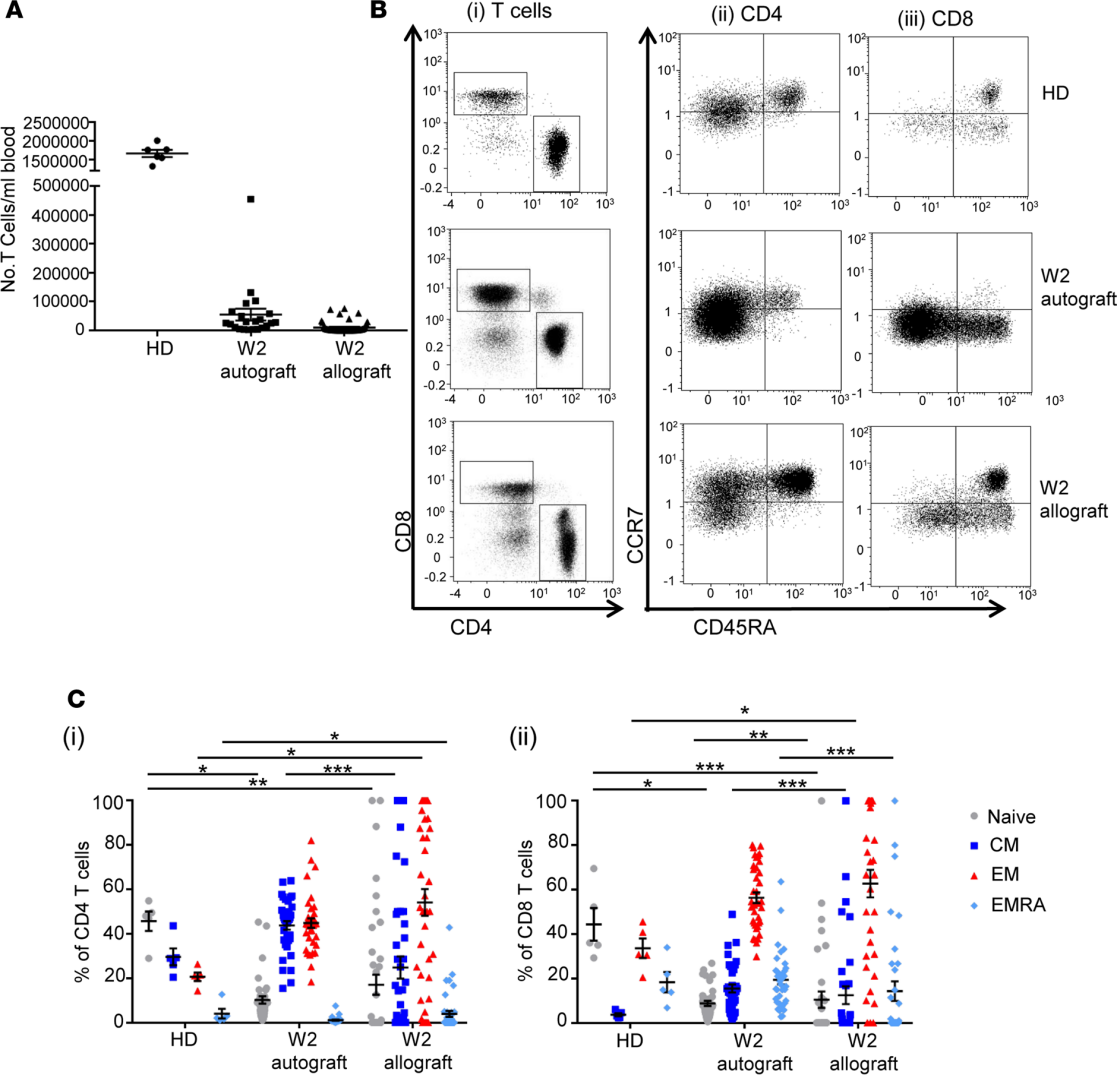
Circulating differentiated allograft and autograft T cells are detectable at week 2 after SCT. (**A**) Number of T cells/ml of whole blood at week 2 after allo-SCT (*n* = 50) and auto-SCT (*n* = 22) and, for comparison, that in healthy donors (HDs; *n* = 6). Error bars represent SEM. (**B**) Representative flow cytometric plots demonstrating the presence of CD4 and CD8 T cell populations in an allo-SCT patient and an auto-SCT patient at week 2 after SCT, and in a HD for comparison, that can be further differentiated by their expression of CCR7 and CD45RA (CD4 and CD8). (**C**) Comparison of the relative proportions of the naive, central memory (CM), effector memory (EM), and effector memory RA–positive (EMRA) phenotypes in CD4 and CD8 T cells at week 2 after allo-SCT (*n* = 41 CD4, *n* = 35 CD8) and auto-SCT (*n* = 37) and, for comparison, HDs (*n* = 5). Data were analyzed using a Kruskal-Wallis test with Dunn’s multiple comparisons tests, *P < 0.05, **P < 0.01, ***P < 0.001. Error bars represent SEM.

Blood samples were taken from 51 allograft and 38 autograft patients (Supplemental Table 1; supplemental material available online with this article; https://doi.org/10.1172/jci.insight.97219DS1) at 2 weeks after transplantation, and the number and phenotype of T cells was determined. The mean T cell count was 1.0 × 10^4^ cells/ml within allograft patients and 5.5 × 10^4^ cells/ml in autograft patients, approximately 100-fold less than is found within healthy adults (Figure 1A). To determine the T cell differentiation state, we analyzed CCR7 and CD45RA expression to identify naive, central memory, effector, and “CD45RA-revertant” effector memory subsets (Figure 1, B and C). CD4 and CD8 effector cells in allograft patients were already increased at week 2 and made up 54% and 62% of the repertoire, respectively (*P* < 0.05 compared with healthy donors [HDs]). As anticipated, the proportion of naive T cells was significantly decreased in both patient groups compared with HDs (*P* < 0.05 for autograft CD4 and CD8 cells; *P* < 0.01 for allograft CD4 cells; and *P* < 0.001 for allograft CD8 cells). In line with previous studies (14, 22–24), chimerism analysis of 7 patients demonstrated that 98%–100% of allograft T cells detected at week 2 were of donor origin (data not shown).

### Alloreactive T cell clonal expansions are identifiable by week 2, persist into the subsequent T cell repertoire, and demonstrate selective recruitment into tissue affected by GvHD.

**Figure 2:**
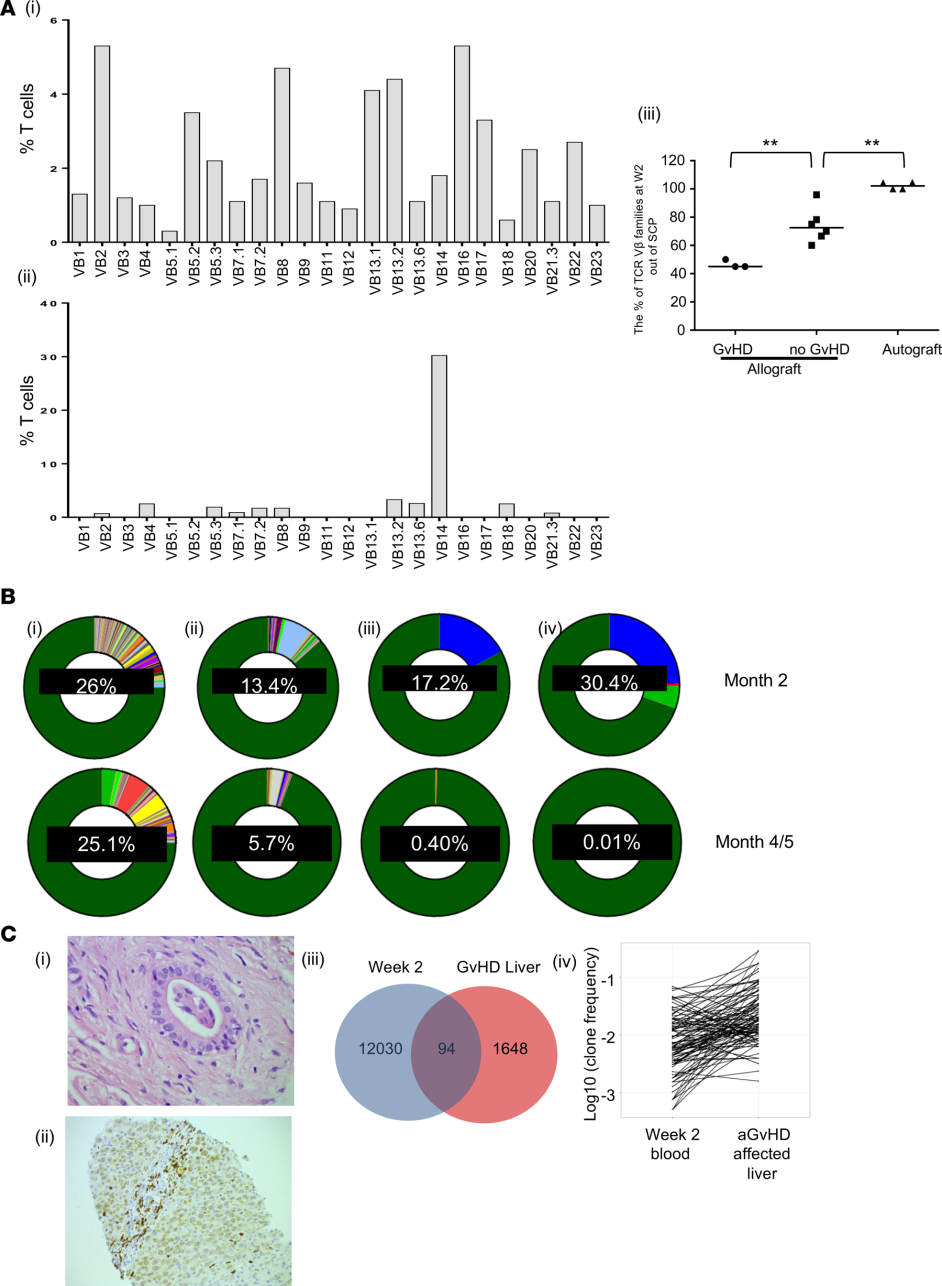
Week 2 T cell clones are implicated in the AIR, persist throughout the period immediately after transplant, and are detectable in GvHD-affected tissues. (**A**) An example of TCRVβ usage by T cells within the stem cell product (SCP; [top left]) and at week 2 (bottom left) after SCT from an allograft patient. Individual TCRVβ families are shown on the *x* axis; the *y* axis shows the percentage of total T cells expressing each individual TCRVβ family. The ratio of SCP and week 2 TCRVβ usage in patients who received autografts, received allografts and did not develop GvHD, and received allografts and did go on to develop GvHD is also shown. The number of TCRVβ families detected at week 2 is represented as a percentage of the number detected within the SCP in paired samples from individual patients. Data were analyzed by 2-tailed, Mann-Whitney *U* test, comparing No GvHD with GvHD and No GvHD with Auto, **P < 0.01. (**B**) Donut charts showing the percentage of the total T cell repertoire at 2 months after and 4–5 months after allo-SCT in 4 patients (left to right) that is attributable to clones identified at week 2 (shown as percentages in the centre of the donuts). Dark green shading represents clones not detectable at week 2. (**C**) H&E-stained section (original magnification, ×60) of liver from a liver aGvHD patient, showing infiltration of bile duct epithelium by atypical lymphocytes, associated with epithelial cell degenerative changes, indicative of aGvHD (top image). A section of liver with CD3 immunostaining from the same patient at (original magnification, ×20), showing infiltration of portal tracts, including bile ducts by CD3-positive lymphocytes (bottom image). Venn diagram showing the number of shared TCRβ chain CDR3 sequences (overlap between blue and pink sets) between week 2 (blue set) and GvHD-affected liver (pink set) in the same patient. Line graph showing the log_10_ frequency of reads for each T cell clone present in the blood at week 2 and in the liver at the point of aGvHD in the same patient.

Having identified circulating T cell populations in the early period after transplant, we went on to examine whether cells present within allograft patients at this stage were implicated in the subsequent development of clinical complications of the AIR. T cell receptor (TCR) Vβ family expression was assessed using FACS on T cells from paired stem cell product (SCP) and patient samples at week 2 after allograft or autograft transplant. Week 2 T cells from autograft patients retained a polyclonal repertoire. In contrast, the diversity of TCR Vβ family expression after allograft contracted markedly during this period, suggesting expansion of specific T cells clones driven by antigen-specific allorecognition (Figure 2A). Importantly, this pattern was much more pronounced in patients who subsequently went on to develop acute GvHD (aGvHD), an important clinical complication of AIR (*P* < 0.01; Figure 2A, top left).

aGvHD occurred at a median time point of 29 days after transplantation in our cohort, and, as such, we went on to determine the relative persistence of week 2 T cell clones beyond this time point. *TCRVB* CDR3 sequencing was performed in 4 patients to track individual T cell clones from week 2 through to 2 months and 5 months after transplant. Interestingly, clones that were present at week 2 after transplant were detectable at 2 months and 5 months in all 4 donors, demonstrating that T cell clones that emerge early after transplant contribute to the repertoire after transplant in the longer term (Figure 2B).

Based on these data, we were interested to see if T cell clones present at week 2 would show preferential targeting to sites of clinical aGvHD. Therefore, we compared *TCRVβ* CDR3 clonotypes present at week 2 with those identified from the liver biopsy of a patient who developed acute liver GvHD at 96 days after SCT (Figure 2C, left) and the skin biopsy of a patient who developed acute skin GvHD (Supplemental Figure 2). 94 T cell clones were shared between the week 2 blood and liver aGvHD samples (Figure 2C), with the most frequent of these constituting 0.5% of the liver repertoire (Figure 2C, right), increased by 100-fold from a frequency of 0.005% in week 2 blood. Together, these findings indicate that antigen-driven clonal T cells are present by week 2 following allograft and that these may be involved in the development of aGvHD.

### Week 2 allograft T cells are recently activated and undergoing intense proliferation with spontaneous production of cytokines.

**Figure 3:**
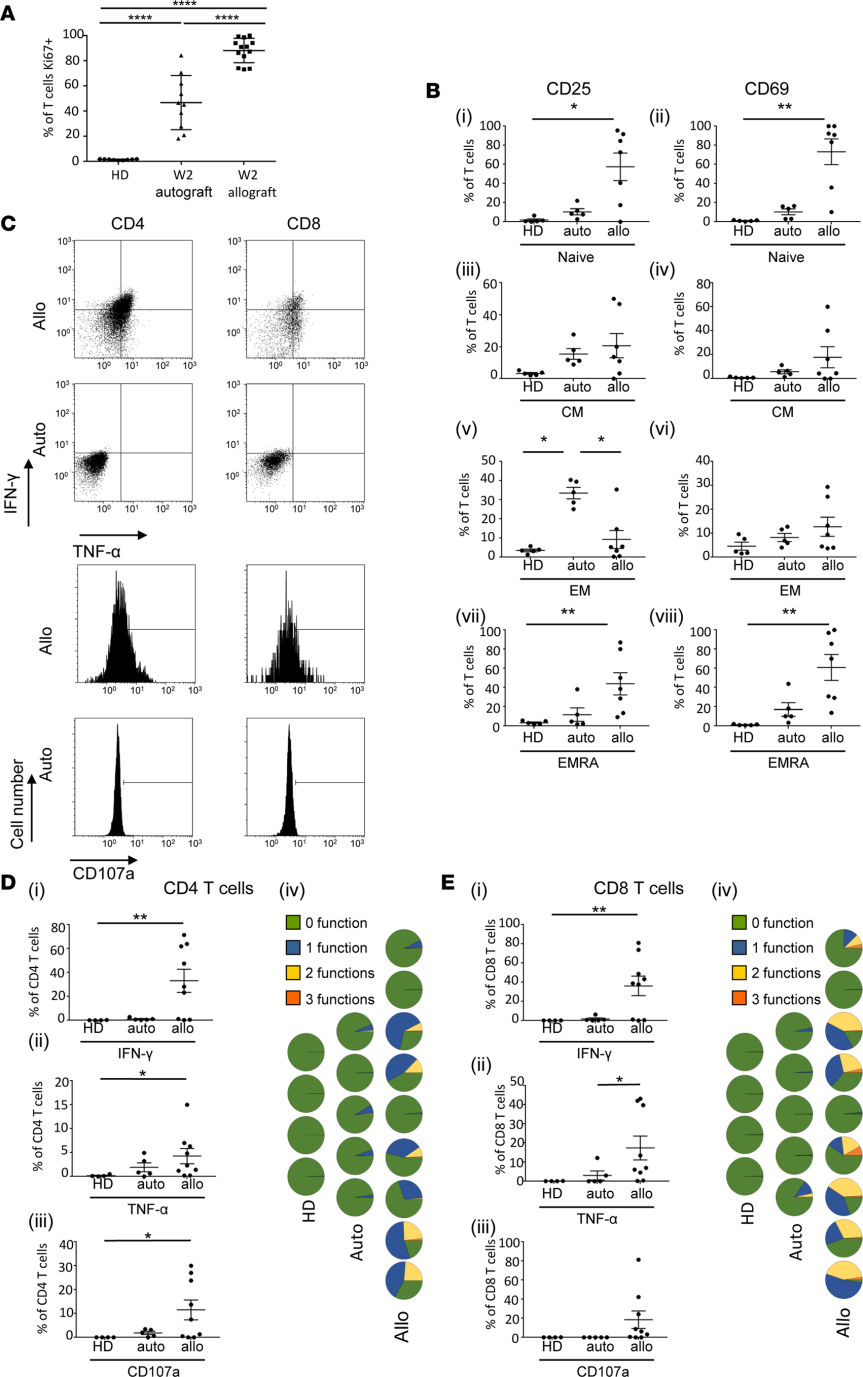
Week 2 T cells from allograft patients have a highly proliferative and activated phenotype. (**A**) Mean Ki-67 expression by T cells at week 2 after allo-SCT (*n* = 13) and auto-SCT (*n* = 10) and, for comparison, healthy donors (HDs; *n* = 9). Error bars represent the SEM; data were analyzed by a 1-way ANOVA with a Tukey’s multiple comparisons test, ****P < 0.0001. (**B**) Expression of CD25 and CD69 by T cells at week 2 after allo-SCT (*n* = 7) and week 2 after auto-SCT (*n* = 5) and that in healthy donors (HDs; *n* = 5) on naive, CM, EM, and EMRA subsets. Data were analyzed using a Kruskal-Wallis test with a Dunn’s multiple comparisons test, *P < 0.05, **P < 0.01. (**C**) Representative FACS plots showing constitutive (i.e., without mitogenic stimulation) IFN-γ and TNF-α production (dot plots) and CD107a expression (histograms) by CD4 and CD8 week 2 T cells from an allograft patient (Allo). Representative plots from an autograft patient (Auto) are shown for comparison. (**D**) Expression of IFN-γ, TNF-α, and CD107a by CD4 week 2 T cells from allograft patients (*n* = 9), autograft patients (*n* = 5), and HDs (*n* = 4). Data were analyzed by a Kruskal-Wallis test with a Dunn’s multiple comparisons test, *P < 0.05, **P < 0.01. Polyfunctionality plots showing number of functions exhibited by CD4 T cells from each allograft, autograft, and healthy donor. Each pie chart represents 1 individual. (**E**) Expression of IFN-γ, TNF-α, and CD107a by CD8 week 2 T cells from allograft patients (*n* = 9), autograft patients (*n* = 5), and HDs (*n* = 4). Data were analyzed by a Kruskal-Wallis test with a Dunn’s multiple comparisons test, *P < 0.05; **P < 0.01. Polyfunctionality plots showing number of functions exhibited by CD8 T cells from each allograft, autograft, and healthy donor. Each pie chart represents 1 individual.

Our TCR sequencing data showed that week 2 T cell clones could persist and may play a role in the allogeneic response beyond the early time period after transplant. To examine whether these cells were functionally capable of involvement in the AIR, we analyzed their phenotype and function and compared these findings to week 2 autograft T cells and cells from HDs. An analysis of Ki67 expression demonstrated that >80% of allograft T cells were proliferating. While a high degree of proliferation is to be expected in such a lymphopenic environment, lymphocyte count–matched autograft patients had a significantly lower Ki67 expression (Figure 3A, P** < 0.0001), indicating that the allograft proliferation may not be entirely attributable to the profound lymphopenia. In keeping with this, CD25 and CD69 expression showed a gradual decrease, starting with allograft T cells (where they were expressed by up to 98% of the population in the “naive” [CCR7^+^CD45RA^+^] subset) through intermediate levels of expression on autograft T cells to low levels on HDs (Figure 3B). The exception to this was for CD25 expression in the effector memory (CCR7^–^CD45RA^–^) subset, where autograft T cells had significantly greater levels of expression (Figure 3B, P** < 0.05) compared with both allograft and HD samples.

Next, we analyzed allograft and autograft T cells for production of the cytokines IFN-γ and TNF-α and for CD107a expression. Strikingly, both CD4 and CD8 allograft week 2 T cells demonstrated constitutive (i.e., without mitogenic stimulation) production of these cytokines (Figure 3C). Quantitative analysis showed significantly higher levels of IFN-γ, TNF-α, and CD107a in CD4 T cells from allograft patients compared with HDs (Figure 3D) and IFN-γ in CD8 T cells (Figure 3E). Allograft T cells also produced more cytokine that those from autografts, again suggesting that the allogeneic environment produced a T cell activation profile beyond that simply due to the lymphopenia and inflammatory environment associated with the SCT process. Polyfunctional capacity was seen in both allograft CD4 and CD8 lineages, although it was more striking within the CD8 subset (Figure 3, D and E).

### Week 2 allograft T cells display a transcriptional profile of cellular activation and proliferation.

**Figure 4:**
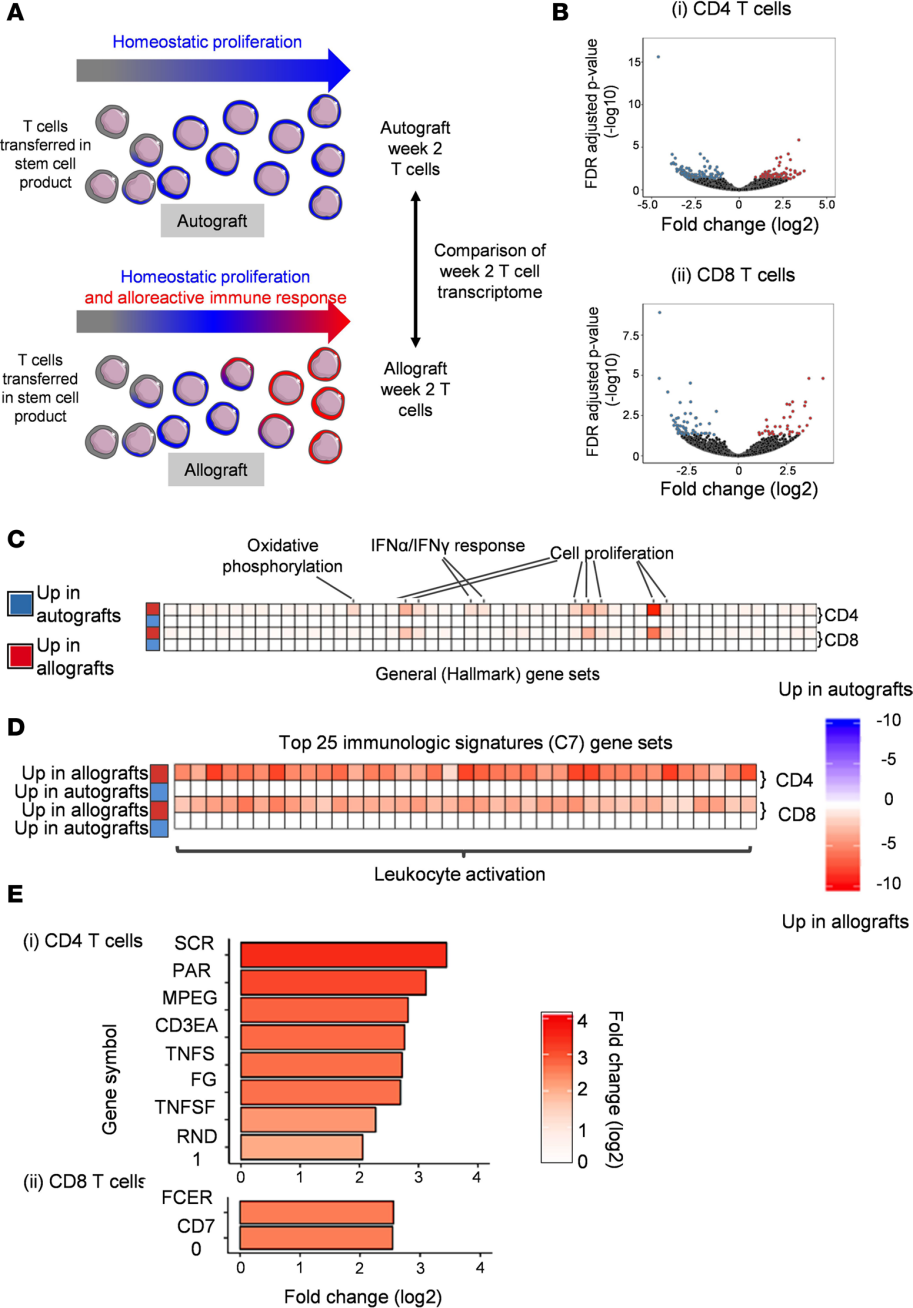
Week 2 allograft T cells have an activated phenotype at the transcriptional level. RNA-Seq was performed on CD4 and CD8 T cells isolated from both the stem cell product (SCP) and peripheral blood at week 2 from 4 allograft and 4 autograft patients. Autograft and allograft samples taken at week 2 were matched for lymphocyte count between patients to control for the influence of homeostatic proliferation due to lymphopenia. (**A**) Representation of experimental design. T cells transferred into an autograft patient will expand under the influence of homeostatic proliferation, whereas donor T cells that are transferred into an allograft patient will proliferate due to the combined influence of homeostatic proliferation and the allogeneic immune response (AIR). The transcriptome of week 2 T cells from both autograft and allograft patients were compared to identify the transcriptional changes associated with the AIR. (**B**) Volcano plots showing differential expression of genes in CD4 and CD8 T cells at week 2 after transplant compared between allograft and autograft patients. Genes more highly expressed in autografts (FDR-adjusted *P* < 0.05) are highlighted in blue, and those more highly expressed in allografts (FDR-adjusted *P* < 0.05) are highlighted in red. (**C**) Heatmap showing all significant (false discovery rate–adjusted [FDR-adjusted] *P* < 0.05) hierarchically clustered GSEA *q* values for the MSigDB “hallmark” gene set. Values shown in blue are higher in autografts than in allografts, and values shown in red are those higher in allografts than in autografts. Columns represent gene sets, and rows represent pairwise comparisons. The first two rows represent FDR-adjusted *P* values for CD4 T cells, and the last 2 rows represent FDR-adjusted *P* values for CD8 T cells. (**D**) Heatmap showing the top 25 significant (FDR-adjusted *P* < 0.05) hierarchically clustered GSEA *q* values for the MSigDB c7 “immunologic signatures” gene sets for CD4 and CD8 T cells, resulting in a total of 37 unique terms. Values shown in blue are those higher in autografts than in allografts, and values shown in red are those higher in allografts than in autografts. Columns represent gene sets, and rows represent pairwise comparisons. The first two rows represent FDR-adjusted *P* values for CD4 T cells, and the last two rows represent FDR-adjusted *P* values for CD8 T cells. (**E**) Bar plots showing log_2_ fold change for selected candidate genes for modulation of the AIR in both CD4 (top) and CD8 (left) T cells. Candidate genes were selected on the basis on at least a log_2_ fold change of 2 and an adjusted *P* value of less than 0.05 in allografts but not autografts and inclusion in the ImmPort database as a gene with an immunological function.

Based on the data we had collected, we hypothesized that by 2 weeks after both autograft and allograft there was a change in T cell phenotype relative to HDs attributable to lymphopenia and the SCT process (Figure 4A). In allograft T cells alone, we hypothesized that there were changes in T cell phenotype (beyond the lymphopenia and SCT-associated changes) attributable to the allogeneic immune response (Figure 4A). To test this, T cells were sorted from autograft and allograft patients (matched for lymphocyte count to control for the influence of homeostatic proliferation) at week 2 and subjected to RNA-sequencing (RNA-Seq) analysis (Figure 4A). This revealed a range of genes that were upregulated in CD4 and CD8 T cells from allograft or autograft patients (Figure 4B; all differentially expressed genes are shown in Supplemental Data 1). Furthermore, gene set enrichment analysis (GSEA), performed using the MSigDB “hallmark” gene set (25), showed that gene sets selectively upregulated in allograft T cells were associated with increased levels of activation and included gene ontology (GO) terms associated with cellular proliferation and the IFN-γ and α responses (Figure 4C; all significantly differentially expressed hallmark GO terms are shown in Supplemental Data 1). Importantly, this profile, which reflected the cellular phenotype that we had observed at week 2, was not seen when the transcriptome of SCP T cells taken from the same allograft and autograft patients was compared. This indicates that the allograft-specific transcriptional signature is established in the period after SCT (all differentially expressed genes in allo-SCT and auto-SCT SCP are shown in Supplemental Data 1).

### Several immune genes are selectively expressed in week 2 allograft T cells and represent potential candidates for early therapeutic immunomodulation.

To further interrogate differences in the transcriptional immune profile between allograft and autograft T cells, we performed GSEA, utilizing the “C7 immunologic signatures” gene set, which is a compendium of gene sets from >5,000 immune studies (26). In concordance with the hallmark GSEA analysis, the top 25 upregulated gene sets in allograft CD4 and CD8 week 2 T cells were again all associated with leukocyte activation (represented in Figure 4D; Supplemental Figure 1B lists the top 25 terms from CD4 cells and the top 25 from CD8 cells, resulting in 37 unique terms; changes in all c7 gene sets are listed in Supplemental Data 1). C7 gene sets that were changed in the allografts compared with the autografts included those associated with antigenic stimulation (2 data sets with GEO accession GSE36476; Supplemental Figure 1B) and those associated with an effector versus memory phenotype (2 GOLDRATH sets; http://software.broadinstitute.org/gsea/msigdb/geneset_page.jsp?geneSetName=GOLDRATH_HOMEOSTATIC_PROLIFERATION; Supplemental Figure 1B). Finally, we utilized this information to identify proteins that might represent potential therapeutic targets for immune modulation of the AIR in the early period after transplant. Specifically, genes were filtered to identify those with a ≥2 log_2_ increase in allografts but not autografts combined with annotation of immunological function in the NIH immunology database and analysis portal (ImmPort) (27). This identified 8 genes within CD4 T cells that are selectively upregulated after allograft, including OX40L (*TNFSF4*) and TWEAK (*TNFSF12*) (Figure 4E). CD70 (*TNFSF7*) and *FCER1G* were identified as upregulated in CD8 allograft T cells (Figure 4E).

## Discussion

Several studies have demonstrated that the AIR is established in the very early period following hemopoietic transplantation. In particular, immunological parameters, such as T cell count and CD4/Treg ratio at week 2, carry prognostic importance (6–11), and early administration of Cy reduces the incidence of GvHD (12, 13). A few studies have examined T cells during this early period after SCT in some detail (17, 18, 28); although, notably, much of the work has been performed after use of the Cy protocol that depletes alloreactive T cells. Therefore, very little is known regarding the phenotype or function of alloreactive T cells at this crucial stage of immune activation.

In the first part of our work, we generated functional evidence for the role of week 2 T cells in the AIR. Specifically, our data indicate that the T cell AIR is detectable at this time point, before development of many of its clinical symptoms. TCR Vβ family analysis demonstrated that autograft T cells retained a polyclonal T cell repertoire during the first 2 weeks of expansion, whereas allograft cells developed an oligoclonal profile by this stage. Importantly, no differences were observed in the rate or severity of infectious complications between auto- and allo-HSCT patients, suggesting that a differential response to infectious agents is not a significant explanation for these differences (Supplemental Figure 4). Moreover, this clonal restriction was much more profound in patients that subsequently developed aGvHD, suggesting that the repertoire changes associated with the AIR were detectable by week 2. This finding is corroborated by other studies demonstrating oligoclonality due to antigen-specific expansions (29) in the absence of thymic export (30) and with reports that identify specific Vβ families as being associated with GvHD (31, 32). The relative oligoclonality in comparison with week 2 autograft T cells lends further weight to this argument. Clearly, a potential limitation of the study is the difference in the conditioning and treatment protocols that are employed for patients undergoing autograft or allograft procedures, including the use of alemtuzumab within allograft patients, which depletes both T cells and additional immune subsets. Lymphocyte count–matched autograft and allograft samples were used to mitigate against these differences, as far as possible, and candidate target gene analysis focused specifically on immune genes.

Previous studies have indicated that T cell populations that become highly activated after SCT may fail to enter the memory pool due to intense and persistent activation by antigen on nonhematopoietic cells (29). As such, it was important to assess if clones present at week 2 were recruited into the longer-term T cell repertoire: our data suggest that a substantial proportion of the long-term repertoire is already established at week 2. In addition, we could show that T cell clones identified at week 2 were present 14 weeks later within liver aGvHD samples, with some clones showing up to 100-fold selective recruitment into tissue. Importantly, the functional capacity of T cells at day 14 to recognize host tissue was measured by ELISPOT analysis, and alloreactive T cells were indeed detectable at week 2 after transplant (Supplemental Figure 3).

Our functional assays identified high levels of proliferation, constitutive production of inflammatory cytokines and upregulation of markers of activation by week 2 T cells from allograft patients. Our findings show that T cells are undergoing intense proliferation in the early after SCT period, with Ki67 expression observed on nearly all peripheral T cells. It is known that homeostatic proliferation is a potent stimulus for T cells (20, 21), but it is noteworthy that Ki67 expression was much lower in autograft recipients at this time point. As such, these data suggest that antigen-specific allorecognition is already well established at week 2. This proliferative phenotype was mirrored by the high level of expression of the activation markers CD69 and CD25 on both CD4 and CD8 allograft T cells. This was particularly striking on cells with a phenotypically “naive” phenotype, where both CD69 and CD25 were expressed by most of the population (Supplemental Figure 5). Naive donor T cells, which are activated by recognition of host peptide, play an important role in generation of the AIR (30, 31), and these findings would suggest that this CD45RA^+^CCR7^+^CD69^+^CD25^+^ population represents cells that have undergone recent alloreactive recognition. A clinical trial of naive T cell depletion has indicated that the naive population is not entirely responsible for development of the acute AIR (33). We also observed that CD69 and CD25 were upregulated on memory and effector T cells at day 14 after transplant, although it is not possible to determine if this represents recently activated naive T cells or memory subsets. Previous studies have demonstrated that, while donor memory and effector T cells play a variable and potentially less important role compared with naive T cells in the induction of aGvHD (32, 34–36), these cells may be involved in the graft-versus-leukemia response, which is also part of the AIR (36–39). CD69 expression was much lower on T cells following autograft, suggesting that this protein could be a useful marker of cells that have undergone antigen-specific allorecognition. Indeed, CD69 has been used for therapeutic pretransplant depletion of alloreactive T cells following culture with host cells in vitro (40), and our findings indicate that this could also be of value at week 2.

A particularly striking feature of our ex vivo analyses was the finding that many T cells from allograft patients display spontaneous production of cytokines without the need for stimulation with mitogens. This is a highly unusual feature, although it has been reported previously in leukemia cells and T cells infected with human T cell lymphotropic virus (41–44). Again, this phenotype was seen in only a small minority of T cells from autograft patients, which indicates that the alloreactive stimulation that occurs following allogeneic transplantation is a key requirement. Cytokine secretion could play a significant role in the development of GvHD, and this observation may indicate that anti-cytokine therapy could be of clinical utility at this early stage. Collectively, these findings provide strong evidence that the T cell alloimmune response is detectable in the early period after transplant, well before the development of associated clinical symptoms.

This characterization of the developing AIR raises the possibility of potential early therapeutic intervention to manipulate the clinical outcome of the transplant. As such, we examined the transcriptome of week 2 CD4 and CD8 allograft T cells using RNA sequencing. An important control was that this information was compared with similar analysis of samples from autograft patients with matched lymphocyte counts, enabling us to control for the effects of homeostatic proliferation on T cell phenotype. GSEA analysis using “hallmark” and “immune” gene sets demonstrated that week 2 allograft T cells had high level expression of genes associated with the cell cycle and T cell activation, revealing processes that are triggered by allorecognition rather than homeostatic expansion, in line with murine models (20). Interestingly, this pattern was similar across CD4 and CD8 cells, indicating involvement of both major lineages in the alloreactive response. Transcriptional analysis was also used to identify genes that might represent potential therapeutic targets for modulation of the AIR. Several immune genes were upregulated in CD4 T cells, including two members of the TNF superfamily, *TNFSF4* and *TNFSF12*. *TNFSF4* encodes OX40L, whose primary expression is inducible on antigen-presenting cells, but which has also been demonstrated as a potent stimulatory ligand on subpopulations of activated T cells and may serve to mediate autocrine regulation through OX40/OX40L ligation (45). *TNFSF12* encodes the TWEAK cytokine ligand, an inducer of apoptosis whose excessive expression has been associated with chronic inflammation and fibrosis (46). These findings implicate CD4 T cells as major effector cells in the immunopathology associated with AIR and are supported by our finding of high levels inflammatory cytokine production by CD4 cells in the early period after SCT. Additional upregulated genes encoded for a range of proteins, including the secernin protein, SCRN1; perforin-like protein, MPEG1 (47); PARD3; the CD3-associated enzyme, CD3EAP; the tyrosine kinase, FGR; and the GTPAse, Rnd1. Two potential target genes were upregulated within the CD8 subset: the TNF family member CD70 and *FCER1G*. CD70 is expressed on highly activated T cells, and engagement with its ligand CD27 induces T cell proliferation and cytotoxicity (48) and is believed to play a critical role in the initiation of primary CD8 T cell responses (49). Genetic polymorphism within CD70 has been identified as a risk factor for GvHD, and murine models have demonstrated that CD70 blockade prevents rejection of cardiac allografts (50). CD70 blockade in the early period after transplant may therefore represent an opportunity to suppress GvHD, and this potential is further supported by the identification of CD70 as a direct therapeutic target on tumors (51). FCER1G is a high-affinity receptor of IgE, and its role on CD8 T cells is currently unclear. Clearly, a potential limitation of the study is the differences in treatment that autograft and allograft patients receive. We have tried to mitigate against these differences as far as possible by using lymphocyte count–matched autograft and allograft samples and by using immune genes specifically for our candidate target gene analysis. In addition, importantly, the differences in the allograft and autograft week 2 T cell transcription profiles were not reflected in the transcriptome of the SCP T cells from the same autograft and allograft patients.

**Figure 5:**
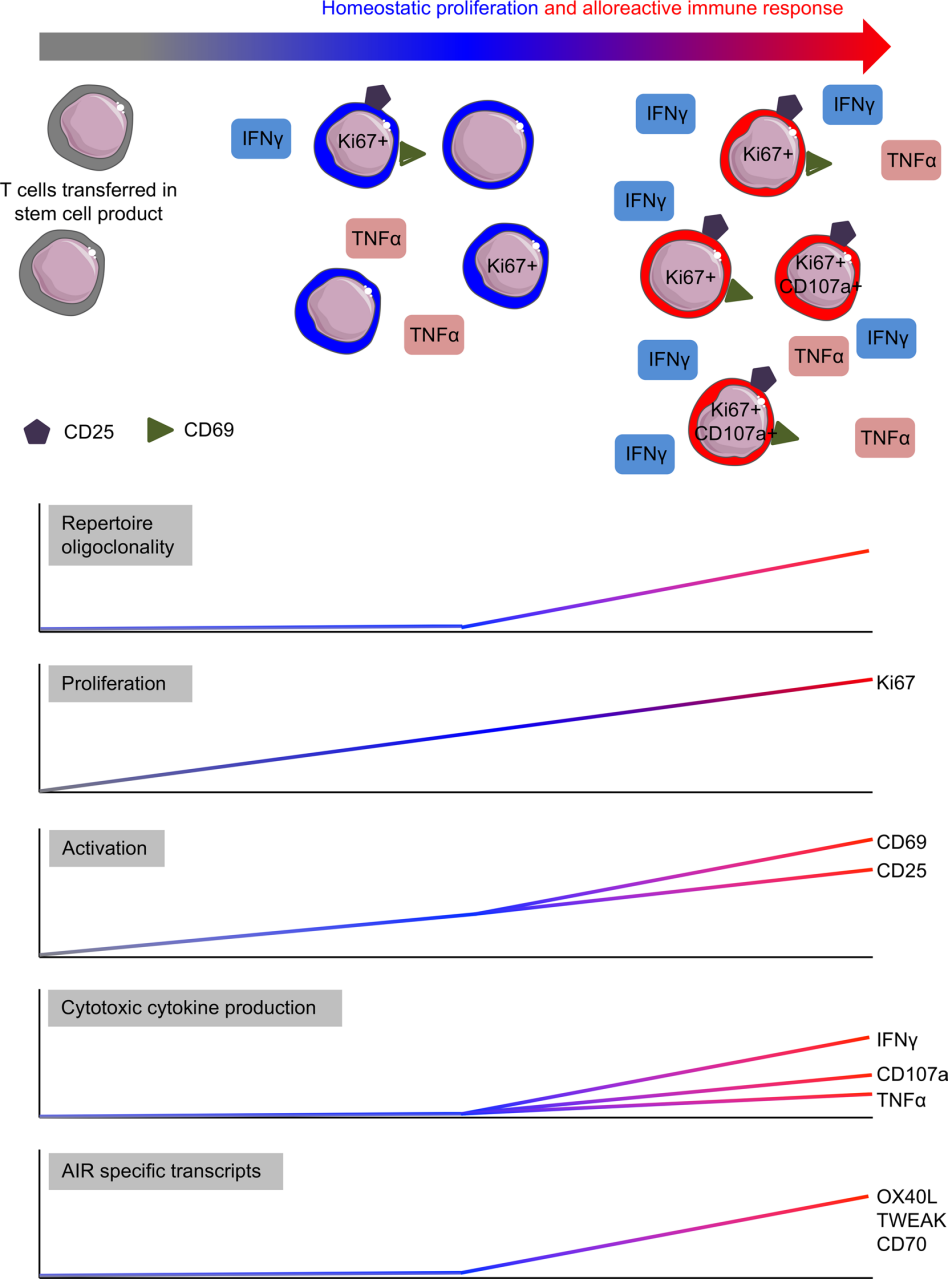
Week 2 allograft T cells have a unique phenotype. Schematic based on the data generated in this study. It shows the effect of homeostatic proliferation (represented in blue) followed by the alloreactive immune response (represented in red) on repertoire, proliferation, expression of activation markers, spontaneous production of cytotoxic cytokine, and the AIR-specific transcriptome of T cells after allo-SCT.

AIRs are critical determinants of clinical outcome following human transplantation. These studies use the approach of comparing autograft and allograft week 2 T cells to isolate and specifically characterize the early T cell AIR, demonstrating that this response is established very early after exposure of the immune system to allogeneic tissue. Our data describe a phenotype associated with week 2 alloreactive T cells, high levels of proliferation, expression of early activation markers, and spontaneous cytotoxic cytokine production (summarized in Figure 5), reflected in the transcriptome of the whole T cell population. Further detailed analysis of the early alloreactive T cell population using this methodology has the potential to uncover a range of novel therapeutic targets.

## Methods

### Patient cohort

Patients undergoing allogeneic or autograft SCT for hematological malignancies at the Queen Elizabeth Hospital Birmingham were enrolled on the study following full written informed consent (05/Q2707/175). All patient details are shown in Supplemental Table 1.

For GvHD prophylaxis, patients received either cyclosporine only or a combination of cyclosporine and methotrexate. Patients were monitored clinically for 120 days after SCT and grouped according to the development of clinically confirmed aGvHD during this period. Classification of aGvHD was carried out according to the NIH consensus criteria, which classifies patients with either classical aGvHD or delayed aGvHD (where typical features of aGvHD present beyond 100 days after transplant, often in the context of withdrawal of immune suppression) (52). Patients who exhibited grade II–IV classical aGvHD were classified into the GvHD-positive group. Treatments received by each individual patient are indicated in Supplemental Table 1.

### Sample collection

#### Peripheral blood.

10–50 ml peripheral blood was collected at week 1 and week 2 after transplant. Blood was taken into sodium heparin tubes for flow cytometric analysis or into EDTA tubes for Vβ TCR repertoire assessment or for chimerism analysis. For all subsequent experiments, fresh blood-derived T cells were used.

#### SCP.

Following receipt of the stem cell graft by patients, residual graft T cells from both allograft and autograft patients were retrieved from the stem cell bag by flushing with PBS. Stem cells were frozen in FCS containing 10% DMSO and stored at –80°C for later use.

### Phenotypic analysis of T cells

Peripheral blood mononuclear cells (PBMCs) were isolated by density gradient centrifugation. Mononuclear cells were surface stained with the appropriate, pretitrated antibodies (see below) on ice for 20 minutes, protected from light, and compared with unstained and fluorescence minus one controls.

T cell subsets were identified using the following monoclonal antibodies: αβTCR-Pacific blue (Biolegend, clone IP26), CD4-APC/Cy7 (BD Biosciences, clone RPA-T4), and CD8-V500 (BD Biosciences, clone RPA-T8) or CD8 PerCPVio700 (Miltenyi Biotech). Naive and memory populations were defined with CD45RA-AF700 (Biolegend, clone HI100) and CCR7 (APC or FITC) (R&D Systems, clone 150503) expression. CD25-PE/Cy7 (BD Biosciences, clone M-A251) and CD69-FITC (BD Biosciences, clone FN50) were included for analysis of activation-associated molecule expression. Propidium iodide (PI) was added immediately prior to acquisition to exclude dead cells. Data were acquired on an LSR-II flow cytometer and analyzed using FACSDiva software (both BD Biosciences) or acquired on a Gallios followed by analysis using Kaluza software (both Beckman Coulter). The lymphocyte population was identified by forward scatter/side scatter dot plots, and live T cells were then identified from this population by gating on PI^–^αβTCR^+^ cells. Clinical counts (lymphocytes per ml whole blood) were provided by the Queen Elizabeth Hospital for each patient.

### Chimerism analysis

PBMCs were isolated from whole blood taken into EDTA at day 6–8 and day 12–14 after allo-SCT. T cells expressing αβTCR were purified by magnetic separation by staining with αβTCR-PE (Biolegend, clone IP26) followed by incubation with an anti-PE bead and purification on an MS column (Miltenyi Biotech). Purified T cells (89%–93% purity) were analyzed by microsatellite analysis at the West Midlands Regional Genetics Laboratory, Birmingham Women’s NHS Foundation Trust, Birmingham, United Kingdom. DNA was extracted from T cells using the Qiagen EZ1 tissue kit according to the manufacturer’s instructions using a Qiagen BioRobot EZ1 or EZ1 Advanced. A panel of 16 polymorphic microsatellite markers (D4S2366, D2S1338, D13S742, Penta D, D10S2325, D12S391, D18S51, D13S634, D18S535, D6S957, D21S11, D18S386, D13S305, D21S1437, FGA, and Penta E) was PCR amplified from donor samples and recipient samples before and after transplant. PCR amplification was performed using fluorescently labeled primers (ABI) in a single-reaction tube (95°C for 15 minutes, 94°C for 30 seconds, 58°C for 1 minute 30 seconds, 72°C for 1 minute 30 seconds) for 25 cycles at 72°C for 10 minutes). Standard reaction conditions were amplified from the 25 ng template DNA; however, for αβTCR T cells the quantity of template DNA used varied from 12–25 ng, depending on the starting DNA concentration. Following PCR amplification, products underwent capillary electrophoresis using an ABI 3130 Genetic Analyzer. Peak labeling was performed using ABI GeneMapper software. Donor and pretransplant recipient traces were assessed, and informative allelic configurations were identified (53), followed by data export and chimerism analysis using an Excel spreadsheet developed in-house. The percentage of donor DNA in a sample was calculated from the relative peak heights and peak areas for donor and recipient from a minimum of two markers with an informative allelic configuration. In-house corrections for stutter and preferential amplification of shorter alleles were applied. Results were reported as the median value of the markers assessed. Importantly, typical sensitivity is 1%–2%; therefore, when 100% donor chimerism is reported, this does not exclude up to 2% host DNA. Full donor chimerism is defined as ≥98% donor, although if host alleles are evident this would always be reported as mixed chimerism.

### Analysis of Vβ family usage by flow cytometry

Vβ family usage was assessed using a TCR Vβ Repertoire Kit (Beckman Coulter) in combination with CD3-PC5 (Beckman Coulter, clone UCHT1). Cells were analyzed using an LSR-II flow cytometer (BD Biosciences)

### DNA isolation and TCRB CDR3 analysis

Genomic DNA was isolated from MACS-sorted (Miltenyi Biotech) T cells (>95% purity) using the QIAamp DNA mini kit as per the manufacturer’s protocol. Sequencing of the TRB locus was performed at Adaptive Biotechnologies using the immunoSEQ assay at survey-level resolution. Initial analysis of sequence reads was performed at Adaptive Biotechnologies using the ImmunoSEQ software tool (Adaptive Biotechnologies). The TCRB CDR3 region was defined as previously described (54). Sequences that did not match CDR3 sequences were removed from the analysis. Sequences that included insertions or deletions resulting in frame shifts or contained premature stop codons were discarded from the analysis, as were sequences that did not resolve to a V, D, and J family.

### Ex vivo analysis of cytokine production and CD107a expression

For analysis of cytokine production and CD107a degranulation, PBMCs were resuspended in RPMI containing 100 U/ml penicillin and 100 μg/ml streptomycin, 2 mM L-glutamine, and 10% FCS and incubated with monensin (1.25 μg/ml and anti-CD107a FITC (BD Biosciences, clone H4A3) for 4 hours. Following incubation, cells were stained with the phenotyping antibodies listed above. They were then fixed (2% PFA), permeabilized (1% Saponin), and incubated with the monoclonal antibodies TNF-α-PE-Cy7 (Ebioscience, clone MAb11) and IFN-γ-AF700 (Biolegend, clone 45.B3) for 20 minutes at room temperature. Cells were analyzed using an LSR-II flow cytometer (BD Biosciences).

### RNA extraction, sequencing, and alignment

RNA was extracted from FACS-sorted T cells (>99% purity) from 4 allograft and 4 autograft patients (Supplemental Figure 1A) using the Qiagen RNeasy micro kit, according to the provided protocol. Extracted RNA was then submitted to Oxford Gene Technologies for RNA-Seq on a HiSeq 2000/2500 as 100–base paired end reads. The preprocessing of RNA-Seq data included trimming of Fastq reads with Prinseq (55) followed by mapping of the reads to the hg19 reference genome using Star Aligner (56). After successful alignment to the reference genome, the reads were quantitated using HTSeq (57). The RNA-Seq data are available in the GEO database (https://www.ncbi.nlm.nih.gov/geo/) under accession GSE111377.

### Differential expression and GSEA

DESeq2 (version 1.14.1) (58) was used to perform differential expression analysis and to compute fold changes. T cells were compared between allograft and autograft SCP samples and across allograft and autograft week 2 samples. CD4 and CD8 T cell contrasts were performed independently, resulting in a total of 4 pairwise comparisons. Genes were considered differentially expressed at an FDR-adjusted *P* value threshold of less than 0.05.

For GSEA, genes with a log_2_ fold change of 0 were removed to avoid rank ties. Ensembl identifiers were converted to Entrez identifiers using biomaRt (version 2.30.0). GAGE (version 2.24.0) was used for all GSEA analyses and to retrieve GO annotations. For further analysis, the MSigDB (v5.2) hallmark (25) and c7 immunologic signatures (26) were used. For display in Figure 4C and Figure 5, the most significant differentially regulated pathways were hierarchically clustered based on the lower and greater FDR-adjusted *P* values for each of the 4 pairwise comparisons.

### Candidate gene selection

Candidate genes were selected from the differentially expressed set of genes (FDR-adjusted *P* < 0.05), with the additional criteria that each gene shows an absolute fold change of above 2 and that each gene is listed in the ImmPort database (27) as having an immunological function. Candidate gene selection was performed independently for CD4 and CD8 T cells.

### Statistics

Univariate analyses comparing two groups were performed using a Mann-Whitney *U* test. For analyses involving more than two groups, either a 1-way ANOVA with Tukey’s multiple comparison test or a Kruskal-Wallis with Dunn’s multiple comparisons test was used. Tests were performed using GraphPad Prism (version 7.02; GraphPad Software Inc.). For TCR CDR3 region analysis, all plots and statistical analyses were performed in R version 3.2.0.

### Study approval

Patients undergoing allogeneic or autograft SCT at the Queen Elizabeth Hospital Birmingham were enrolled in the study following full written informed consent (05/Q2707/175). The ethics of this study have been approved by the NHS National Research Ethics Service (NRES) Committee, West Midlands, South Birmingham, United Kingdom.

## Author contributions

CFI, SAE, and JEC designed research, performed research, performed statistical analysis, analyzed and interpreted data, and wrote the manuscript; NJD and ASO performed statistical analysis and interpretation of the data; TR, HP, MS, YLTC, KV, and JZ performed research and assisted in data interpretation; JN collected clinical data; SN, FK, JN, and RAK consented the patients and collected clinical samples and clinical outcome data; CC assisted in data interpretation; RM assisted in data interpretation and manuscript preparation; and PM designed research, interpreted data, and wrote the manuscript.

## Supplementary Material

Supplemental data

Supplemental Table 1

## Version 1 05/17/2018

Electronic publication
