# Supplementary material for: Unique features and clinical importance of acute alloreactive immune responses
Source: JCI Insight. 2018 May 17;3(10):e97219. doi: 10.1172/jci.insight.97219 (PMC6012511; doi:10.1172/jci.insight.97219)
Supplement: Supplemental data [file jciinsight-3-97219-s006.pdf]

Patient code	Diagnosis	Age at transplant	Donor	Conditioning	GVHD prophylaxis	GvHD II-IV	Tissue affected	Grade	Acute GvHD Classification	Time to GvHD (days)	GvHD treatment	Patient CMV status	Donor CMV status
1_Allo	AML	61	MUD	Flu/Melph/Alem	CSA only	No			N/A			Pos	Pos
2_Allo	CLL	68	SIB	Flu/Melph/Alem	CSA only	No			N/A			Pos	Neg
3_Allo	MDS	58	MUD	Flu/Melph/Alem	CSA only	No			N/A			Pos	Neg
4_Allo	AML	53	SIB	Flu/Melph/Alem	CSA only	No			N/A			Pos	Neg
5_Allo	ALL	64	MUD	Flu/Melph/Alem	CSA only	Yes	Skin	II	Classic aGvHD	43	Pred	Pos	Neg
6_Allo	HL	55	SIB	Flu/Melph/Alem	CSA only	No			N/A			Pos	Neg
7_Allo	AML	51	MUD	Flu/Melph/Alem	CSA only	No			N/A			Pos	Neg
8_Allo	AML	62	MUD	Flu/Melph/Alem	CSA only	No			N/A			Pos	Pos
9_Allo	CLL	53	SIB	Flu/Melph/Alem	CSA only	Yes	Liver	IV	Classic aGvHD	20	Methyl Pred (IV) and Pred	Pos	Neg
10_Allo	CLL	59	MUD	Flu/Melph/Alem	CSA only	No			N/A			Pos	Neg
11_Allo	AML	37	MUD	TBI/CY	CSA + MTX	No			N/A			Pos	Neg
12_Allo	AML	21	MUD	TBI/CY	CSA + MTX	Yes	Lower Gut	II	Classic aGvHD	14	Methyl Pred (IV) then oral Pred	Neg	Neg
13_Allo	CLL	44	SIB	Flu/Melph/Alem	CSA only	No			N/A			Pos	Pos
14_Allo	ALL	21	MUD	Cy/TBI	CSA + MTX	Yes	Skin	II	Classic aGvHD	55	Methyl Pred (IV)	Pos	Neg
15_Allo	AML	70	MUD	Flu/Melph/Alem	CSA only	No			N/A			Pos	Neg
16_Allo	GS	66	MUD	Flu/Melph/Alem	CSA only	Yes	Skin	II	Classic aGvHD	29	Methyl Pred (IV)	Pos	Pos
17_Allo	HL	48	MUD	Flu/Melph/Alem	CSA only	Yes	Skin	II	Classic agvHD	45	Methyl Pred (IV)	Pos	Neg
18_Allo	AML	66	MUD	Flu/Melph/Alem	CSA only	Yes	Lower Gut	II	Delayed aGvHD	157	Methyl Pred (IV)	Pos	Neg
19_Allo	AML	23	MUD	Cy/TBI	CSA + MTX	No			N/A			Neg	Pos
20_Allo	AML	44	MUD	Cy/TBI	CSA + MTX	No			N/A			Pos	Pos
21_Allo	AML	54	SIB	Flu/Melph/Alem	CSA only	No			N/A			Neg	Neg
22_Allo	ALL	44	MUD	TBI/Cy	CSA + MTX	Yes	Lower Gut	II	Classic aGvHD	24	Methyl Pred (IV)	Neg	Neg
23_Allo	AML	58	SIB	Flu/Melph/Alem	CSA only	Yes	Skin	II	Classic aGvHD	36	Methyl Pred (IV)	Neg	Pos
24_Allo	AML	44	MUD	Flu/Melph/Alem	CSA only	No			N/A			Pos	Neg
25_Allo	MDS	57	SIB	Flu/Melph/Alem	CSA only	No			N/A			Neg	Pos
26_Allo	MF	49	SIB	Flu/Melph/Alem	CSA only	No			N/A			Neg	Neg
27_Allo	AML	62	SIB	Flu/Melph/Alem	CSA only	No			N/A			Neg	Neg
28_Allo	MF	44	MUD	Flu/Melph/Alem	CSA only	No			N/A			Pos	Neg
29_Allo	Double Hit lymphoma	46	MUD	Beam/Alem	CSA + MTX	Yes	Skin	II	Delayed aGvHD	134	Pred	Neg	Pos
30_Allo	AML	52	SIB	Flu/Melph/Alem	CSA only	No			N/A			Neg	Pos
31_Allo	NHL	51	MUD	Flu/Melph/Alem	CSA only	No			N/A			Neg	Neg
32_Allo	NHL	57	MUD	Flu/Melph/Alem	CSA only	No			N/A			Pos	Pos
33_Allo	NHL	35	SIB	Beam/Alem	CSA + MTX	Yes	Skin and Gut	II	Classic aGvHD	14	Pred (skin) systemic steroids Methyl Pred (gut)	Pos	Pos
34_Allo	AML	45	MUD	Flu/Melph/Alem	CSA only	No			N/A			Neg	Neg
35_Allo	AML	43	MUD	Flu/Melph/Alem	CSA only	No			N/A			Pos	Pos
36_Allo	MDS	67	MUD	Flu/Melph/Alem	CSA only	Yes	Skin	II	Delayed aGvHD		Topical steroids, increased CSA	Pos	Neg
37_Allo	AML	64	MUD	Flu/Melph/Alem	CSA only	No			N/A			Pos	Neg
38_Allo	NHL	50	MUD	Beam/Alem	CSA only	Yes	Lower Gut	II	Classic aGvHD	12	Pred	Neg	Neg
39_Allo	AML	63	SIB	Flu/Melph/Alem	CSA only	No			N/A			Pos	Neg
40_Allo	B-ALL	54	MUD	Flu/Melph/Alem	CSA only	No			N/A			Pos	Neg
41_Allo	AML	61	MUD	Flu/Melph/Alem	CSA only	Yes	Skin	II	Classic aGvHD	362	Pred	Pos	Neg
42_Allo	AML	62	MUD	Flu/Melph/Alem	CSA only	No			N/A			Pos	Pos
43_Allo	DLBCL	57	MUD	Flu/beam/Alem	CSA only	No			N/A			Neg	Neg
44_Allo	MDS	57	MUD	Flu/Melph/Alem	CSA only	No			N/A			Pos	Pos
45_Allo	AML	65	SIB	Flu/Melph/Alem	CSA only	No			N/A			Pos	Pos
46_Allo	AML	44	MUD	Cy/TBI	CSA + MTX	Yes	Skin	II	Classic aGvHD	50	Pred	Pos	Pos
47_Allo	HL	37	MUD	Flu/Melph/Alem	CSA only	Yes			Delayed aGvHD			Neg	Pos
48_Allo	AML	63	MUD	Flu/Melph/Alem	CSA only	No			N/A			Pos	Pos
49_Allo	AML	21	MUD	Cy/TBI	CSA + MTX	No			N/A			Pos	Pos
50_Allo	Peripheral TNHL	49	SIB	Beam/Alem	CSA only	No			N/A			Neg	Neg
51_Allo	MDS	48	SIB	Flu/Melph/Alem	CSA only	No			N/A			Neg	Pos
52_Allo	AML	25	SIB	Bu/Cy	CSA + MTX	Yes	Liver	III	Classic aGvHD	96			
1_Auto	HL	28	AUTO	BEAM								Pos	
2_Auto	Myeloma	62	AUTO	Melphalan								Neg	
3_Auto	Myeloma	58	AUTO	Melphalan								Pos	
4_Auto	HL	38	AUTO	BEAM								Neg	
5_Auto	Myeloma	51	AUTO	Melphalan								Neg	
6_Auto	Myeloma	65	AUTO	Melphalan								Neg	
7_Auto	NHL	64	AUTO	BEAM								Pos	
8_Auto	Myeloma	65	AUTO	Melphalan								Neg	
9_Auto	Myeloma	70	AUTO	Melphalan								Pos	
10_Auto	Myeloma	40	AUTO	Melphalan								Pos	
12_Auto	HL	54	AUTO	BEAM								Pos	
13_Auto	Myeloma	72	AUTO	Melphalan								Neg	
15_Auto	Myeloma	58	AUTO	Melphalan								Pos	
16_Auto	Myeloma	72	AUTO	Melphalan								Neg	
17_Auto	Myeloma	52	AUTO	Melphalan								Neg	
20_Auto	Myeloma	62	AUTO	Melphalan								Pos	
24_Auto	HL	21	AUTO	BEAM								Pos	
27_Auto	Myeloma	68	AUTO	Melphalan								Neg	
29_Auto	NHL	67	AUTO	BEAM								Pos	
30_Auto	Myeloma	56	AUTO	Melphalan								Neg	
31_Auto	Myeloma	61	AUTO	Melphalan								Neg	
34_Auto	HL	56	AUTO	BEAM								Pos	
36_Auto	Diffuse Large B Cell Lymphoma	66	AUTO	BEAM								Neg	
40_Auto	Myeloma	69	AUTO	Melphalan								Pos	
41_Auto	Myeloma	59	AUTO	Melphalan								Neg	
44_Auto	Systemic Amyloidosis	50	AUTO	Melphalan								Pos	
48_Auto	Myeloma	60	AUTO	Melphalan								Neg	
49_Auto	HL	65	AUTO	BEAM								Pos	
55_Auto	Myeloma	69	AUTO	Melphalan								Neg	
59_Auto	Myeloma	69	AUTO	Melphalan								Pos	
61_Auto	Angioimmunoblastic T-cell Lymphoma	37	AUTO	BEAM								Pos	
62_Auto	Double Hit Lymphoma	67	AUTO	BEAM								Pos	
63_Auto	IgG Myeloma	62	AUTO	Melphalan								Pos	
66_Auto	HL	17	AUTO	BEAM								Neg	
67_Auto	Myeloma	46	AUTO	Melphalan								Neg	
68_Auto	Myeloma	59	AUTO	Melphalan								Pos	
69_Auto	Myeloma	52	AUTO	Melphalan								Neg	
72_Auto	Myeloma	64	AUTO	Melphalan								Pos	

Supplementary table 1. Clinical characteristics of patients included in the study.

The first 52 rows describe allograft patients, remaining rows describe autograft patients. Table includes diagnosis and age at transplant. Characteristics of the transplant are then listed including 'donor type' (characterised as matched unrelated donor (MUD), sibling (SIB) or autograft (AUTO)) and conditioning regimen. Table then includes information relating to GvHD prophylaxis and disease including prophylactic drugs given, development of GvHD (grades II-IV), tissue affected, specific grade, NIHR classification as classic or delayed acute disease, time post SCT to GvHD onset and GvHD treatment strategy. Patient CMV status and donor CMV status at transplant are also included. Acute Myeloid Leukaemia (AML), Chronic Lymphocytic leukaemia (CLL), Myelodysplastic syndrome (MDS), Acute lymphocytic Leukaemia (ALL), Hodgkins lymphoma (HL), Granulocytic Sarcoma (GS), Myelofibrosis (MF), Double Hit lymphoma (DHL), Diffuse large B cell lymphoma (DLB CL) T-cell Non Hodgkins Lymphoma TC-NHL). Matched unrelated donor (MUD), Sibling (SIB), Fludarabine (Flu), Melphalan (Melph), Total body irradiation (TBI), Cyclophosphamide (Cy), Alemtuzumab (Alem), Methotrexate (MTX).

(A)

Allograft or Autograft	Lymphocyte count at week 2/μL whole blood
Allograft	400
Allograft	300
Allograft	400
Allograft	300
Autograft	400
Autograft	300
Autograft	600
Autograft	300

(B)

GSE36476_CTRL_VS_TSST_ACT_72H_MEMORY_CD4_TCELL_OLD_DN
GSE36476_CTRL_VS_TSST_ACT_40H_MEMORY_CD4_TCELL_OLD_DN
GSE15750_DAY6_VS_DAY10_TRAF6KO_EFF_CD8_TCELL_UP
GSE29617_CTRL_VS_TIV_FLU_VACCINE_PBMC_2008_DN
GSE13485_DAY1_VS_DAY21_YF17D_VACCINE_PBMC_DN
GSE21063_WT_VS_NFATC1_KO_8H_ANTI_IGM_STIM_BCELL_UP
GSE24634_TEFF_VS_TCONV_DAY7_IN_CULTURE_UP
GSE33292_WT_VS_TCF1_KO_DN3_THYMOCYTE_DN
GSE10325_LUPUS_CD4_TCELL_VS_LUPUS_MYELOID_DN
GSE21063_CTRL_VS_ANTI_IGM_STIM_BCELL_NFATC1_KO_8H_DN
GSE36476_CTRL_VS_TSST_ACT_72H_MEMORY_CD4_TCELL_YOUNG_DN
GSE39556_CD8A_DC_VS_NK_CELL_MOUSE_3H_POST_POLYIC_INJ_UP
GSE17974_1H_VS_72H_UNTREATED_IN_VITRO_CD4_TCELL_DN
GSE17974_0H_VS_24H_IN_VITRO_ACT_CD4_TCELL_DN
GSE17974_CTRL_VS_ACT_IL4_AND_ANTI_IL12_48H_CD4_TCELL_DN
GSE24634_TREG_VS_TCONV_POST_DAY7_IL4_CONVERSION_UP
GSE36476_CTRL_VS_TSST_ACT_40H_MEMORY_CD4_TCELL_YOUNG_DN
GSE28726_NAIVE_VS_ACTIVATED_CD4_TCELL_DN
GSE13547_2H_VS_12_H_ANTI_IGM_STIM_BCELL_DN
GSE3982_MEMORY_CD4_TCELL_VS_TH2_DN
GSE28726_NAIVE_CD4_TCELL_VS_NAIVE_VA24NEG_NKTCELL_UP
GSE29617_CTRL_VS_DAY3_TIV_FLU_VACCINE_PBMC_2008_DN
GSE33162_UNTREATED_VS_4H_LPS_STIM_HDAC3_KO_MACROPHAGE_DN
GSE13547_CTRL_VS_ANTI_IGM_STIM_BCELL_12H_UP
GSE22886_NAIVE_CD4_TCELL_VS_MONOCYTE_DN
GSE13485_PRE_VS_POST_YF17D_VACCINATION_PBMC_DN
GSE15750_DAY6_VS_DAY10_EFF_CD8_TCELL_UP
GOLDRATH_EFF_VS_MEMORY_CD8_TCELL_UP
GSE17974_CTRL_VS_ACT_IL4_AND_ANTI_IL12_24H_CD4_TCELL_DN
GSE24574_BCL6_LOW_TFH_VS_TCONV_CD4_TCELL_DN
GSE18893_TCONV_VS_TREG_24H_TNF_STIM_UP
GSE14415_INDUCED_TREG_VS_TCONV_UP
GSE29618_BCELL_VS_MDC_DAY7_FLU_VACCINE_DN
GSE13485_DAY1_VS_DAY7_YF17D_VACCINE_PBMC_DN
GSE14415_INDUCED_VS_NATURAL_TREG_DN
GOLDRATH_NAIVE_VS_EFF_CD8_TCELL_DN
GSE5679_CTRL_VS_PPARG_LIGAND_ROSIGLITAZONE_TREATED_DC_UP

Supplementary figure 1. RNA-Seq analysis of transcriptome in SCP and week 2 T cells from allograft and autograft patients.

(A) Table showing the lymphocyte count of each patient used in RNA-Seq study at week 2 post-SCT.

(B) Table showing the the top 25 c7 gene sets from CD4 cells and the top 25 from CD8 cells, resulting in a total of 37 unique terms.

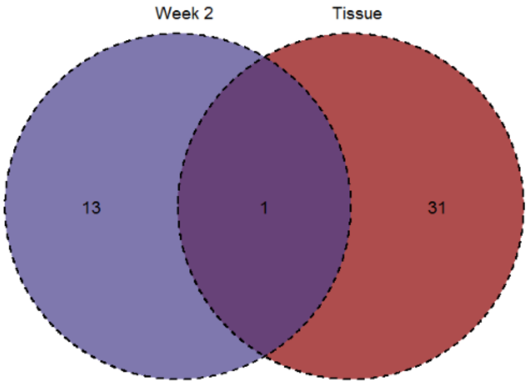

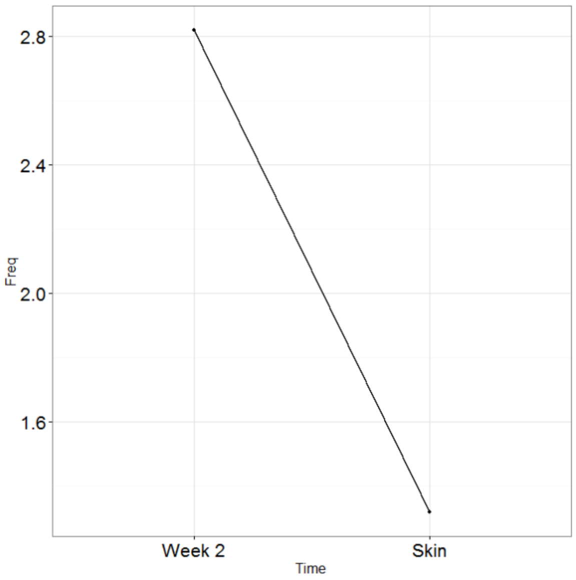

Supplementary Figure 2. **T cell clones at week 2 post HSCT are detectable in GvHD affected tissues.** Venn diagram showing the number of shared TCR beta chain CDR3 sequences between week 2 (blue set) and GvHD Skin (pink set) in the same patient. Line graph shows the log10 frequency of reads for each T cell clone present in the blood at week 2 and in the skin at the point of aGvHD.

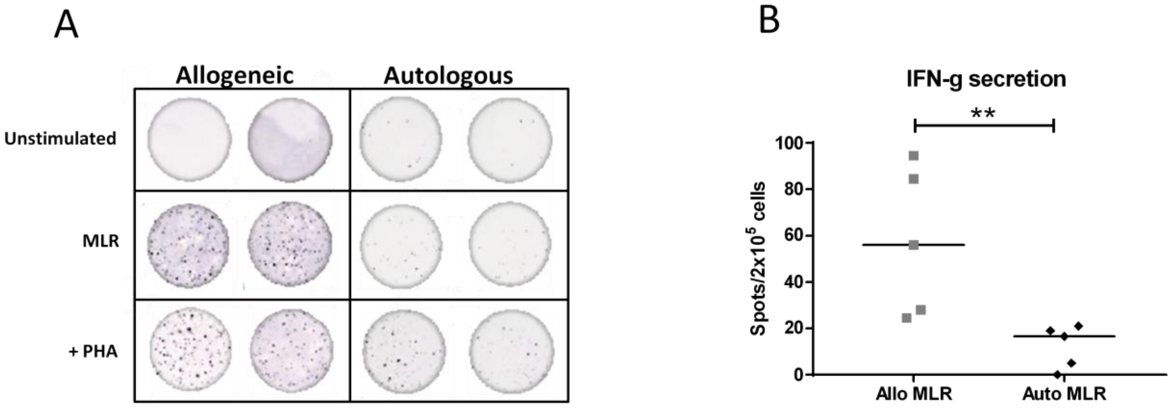

Supplementary Figure 3. Detection of donor derived alloreactive response at 2 weeks after SCT. (A) Representative wells in duplicate depicting Interferon gamma (IFN-γ) secretion by PBMCs isolated from patient at day 14 after allogeneic (autologous) transplant. $1x10^5$ PBMC/well were stimulated either by T cell depleted PBMCs isolated pre-transplant from the patient in a mixed lymphocyte reaction (MLR) or with PHA. (B) IFN-γ release measured in 5 allogeneic and 5 autologous transplant patients as IFN-γ spots/$2x10^5$ cells per well. Statistical comparison was made using Mann-Whitney test, ** depict p value <0.01

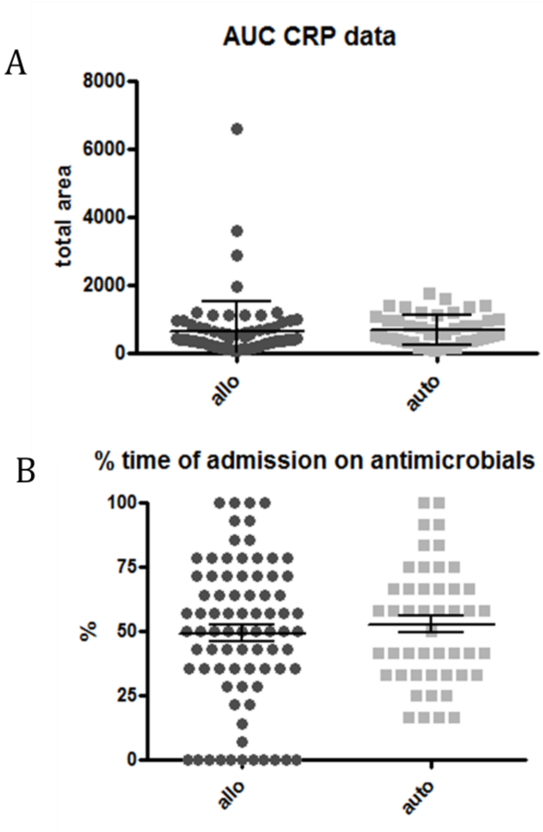

Supplementary Figure 4.There was no difference in the infection rate between the auto and allo-HSCT patients. A. Area under the curve analyses were performed for each patient's CRP levels. Allo-HSCT patients inpatient stays were 14 days on average, while auto-HSCT patient stays were 12 days. There is no difference in the mean AUC suggesting that the inflammatory response early after HSCT was not significantly different in allo versus auto-HSCT. **B**. The percentage of time a patient spent receiving Intravenous antimicrobials throughout their inpatient stay was calculated for all patients. There is no demonstrable difference between allo and auto-HSCT groups.

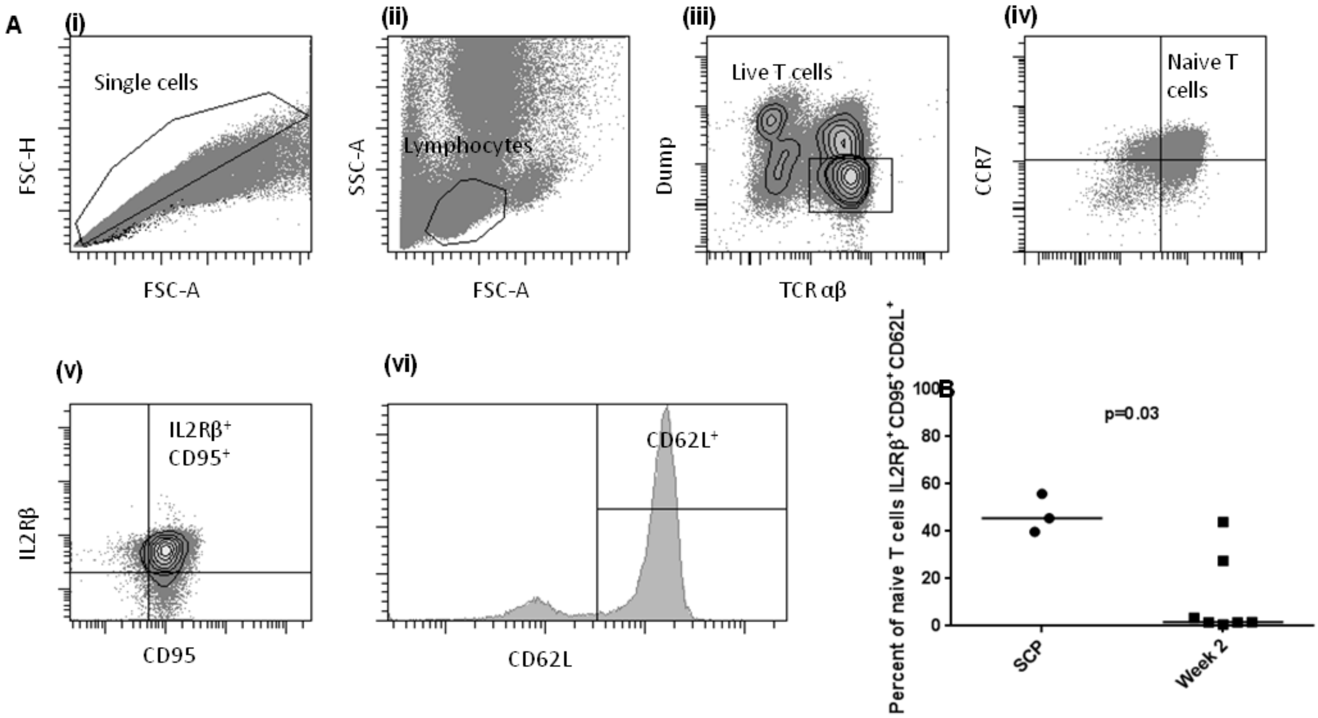

Supplementary Figure 5. Frequencies of memory stem T cells at week 2 and in the stem cell product. Analysis of memory stem T-cells as a proportion of total T cells present in the stem cell product and in matched week 2 peripheral blood of allograft patients. All patients underwent FMC conditioning. Memory stem T-cells are defined as TCRαβ$^+$ CD45RA$^+$ CCR7$^+$ IL2Rβ$^+$ CD95$^+$ and CD62L$^+$. (A) gating strategy: (i) single cells ii) lymphocytes iii) live αβ T-cells iv) CD45RA$^+$ CCR7$^-$ T-cells v) IL2Rβ$^+$CD95$^+$ (vi) CD62L. b) Proportion of memory stem T cells in the stem cell product (SCP) and at week 2 post transplant in matched patient samples. Each line represents one patient; data were analysed by two-tailed, Willcoxon matched-paired sign rank test; *$p<0.05$

Supplementary data 1. Excel spreadsheet listing all data from the RNA-seq analysis of transcriptome in SCP and week2 T cells from autograft and allograft patients.

Tab1: all significantly differentially regulated genes in CD4 and CD8 T cells from SCP T cells (comparing autograft and allograft) and in CD4 and CD8 T cells from week 2 T cells (comparing autograft and allograft). Each row indicates one gene (represented by its ensemble ID). A positive log2 fold change (log2FoldChange) indicates upregulation of the gene in allografts compared to autografts; a negative log2 fold change (log2FoldChange) indicates downregulation of the gene in allografts compared to autografts. T cell type and timepoint are specified in columns H and I. padj: adjusted p value (calculated using the Benjamini-Hochberg procedure).

Tab2: all significantly differentially regulated gene ontogeny (GO) terms in CD4 and CD8 T cells from SCP T cells (comparing autograft and allograft) and in CD4 and CD8 T cells from week 2 T cells (comparing autograft and allograft). Each row indicates one GO term. The columns represent q-values for GSEA tests, comparing allografts vs autografts. The header of each column describes the cell type (CD4 or CD8), the time point (SCP or WK2), and whether each gene set is overrepresented (greater) or underrepresented (less).

Tab3: All significantly differentially regulated MSigDB 'hallmark' gene sets. Each row indicates one gene set. The columns represent q-values for GSEA tests, comparing allografts vs autografts. The header of each column describes the cell type (CD4 or CD8), the time point (SCP or WK2), and whether each gene set is overrepresented (greater) or underrepresented (less).

Tab4: All significantly differentially regulated 'C7 immunological signatures' gene sets. The columns represent q-values for GSEA tests, comparing allografts vs autografts. The header of each column describes the cell type (CD4 or CD8), the time point (SCP or WK2), and whether each gene set is overrepresented (greater) or underrepresented (less).
